# Supplementary material for: A Zero-Valent Sulfur Transporter Helps Podophyllotoxin Uptake into Bacterial Cells in the Presence of CTAB
Source: Antioxidants (Basel). 2023 Dec 22;13(1):27. doi: 10.3390/antiox13010027 (PMC10812762; doi:10.3390/antiox13010027)
Supplement: Supplementary file 1 [file antioxidants-13-00027-s001.zip › antioxidants-2725905-supplementary.pdf]

Supporting Information

A zero-valent sulfur transporter helps podophyllotoxin uptake into bacterial cells in the presence of CTAB

Honglei Liu et al.

Contents

Table S1

Figure S1

Figure S2

Table S1 Oligonucleotide primers used for plasmid construction.

Primers	Nucleotide Sequence	Target gene	Accession number
pBBR-CpYedE1-F	CACACAGGAAACAGCTATGCGGGCACCAGAAATGC	*CpyedE1E2*	WP_011296423
pBBR-CpYedE2-R	TAACAAAATATTAACGCTCAGGCTTGCCGCATGGTGG		WP_011296422
pBBR-CpYedE1-R	TAACAAAATATTAACGCTTATCCCTGCAACTCGGGT	*CpyedE1*	WP_011296423
pBBR-CpYedE2-F	ATTTCACACAGGAAACAGCTATGGCTGCTTTTTTCGC	*CpyedE2*	WP_011296422
pBBR-EcYedE3-F	CACACAGGAAACAGCTATGTTTTCAATGATATTAAGC	*EcyedE3*	WP_000492348
pBBR-EcYedE3-R	TAACAAAATATTAACGCTTAATTTGCCGCAGC		
pBBR-CpYedE3-F	CACACAGGAAACAGCTATGCCCGAGATCGATATCGCCG	*CpyedE3*	WP_011296984
pBBR-CpYedE3-R	TAACAAAATATTAACGCTCAGGCGAATGCCATGCGCT		
pBBR-SmYedE1-F	CACACAGGAAACAGCTATGAGTAAAGGAGAAGAACCACA	*SmYedE12*	WP_017963239
pBBR-SmYedE2-R	TAACAAAATATTAACGCTCACGTCCCTCTGCTCCACA		WP_003546111
pTrc-CpPdo2-F	GACCATGGAATTCGAGCTCATGACACCGACCATGCCAAG	*Cppdo2*	WP_020200642
pTrc-CpPdo2-R	CTGCAGGTCGACTCTAGATCAGAGGGCGTTGAGGG		
pCL1920-Pdo2-F	AGAAAGAGGAGAAATACTAGATGACACCGACCATGCCAAG	*Cppdo2*	WP_020200642
pCL1920-Pdo2-R	TCGTTTTATTTGATGCCTGGTCAGAGGGCGTTGAGGG		
pTrc-CpSqr-F	GACCATGGAATTCGAGCTCATGCAACCCCGCGCCCTC	*CpSqr*	WP_009890921
pTrc-CpSqr-R	CTGCAGGTCGACTCTAGATTATCCCTGCAACTCGGGTGT		
CpYed1-C104S-F	GCGGATCCACCAGCGGGCACGGCGTGTGCGGAATCTCG	*CpyedE1*	
CpYed1-C104S-R	TGCCCGCTGGTGGATCCGCTGGCATAGCGGGTGCCGAT	C104S	
CpYed1-C101S-F	CGCCGGGTTCTcTCCGGGCCCGGCGCTGGTGGCGCTTG	*CpyedE2*	
CpYed1-C101S-R	GGCCCGGAgAGAACCCGGCGATCCCCCAGCCGACGCCG	C101S	
pBBR-Gfp-F	CACACAGGAAACAGCTATGAGTAAAGGAGAAGAACTTT	*egfp*	JQ064510
pBBR-Gfp-R	TAACAAAATATTAACGCTTATTTGTATAGTTCATACATGCCAT		
pTrc-Gfp-F	GACCATGGAATTCGAGCTCATGAGTAAAGGAGAAGAACTT	*egfp*	JQ064510
pTrc-Gfp-R	CTGCAGGTCGACTCTAGATTATTTGTATAGTTCATACATGC		
pCL1920-Gfp-F	AGAAAGAGGAGAAATACTAGATGAGTAAAGGAGAAGAAC	*egfp*	JQ064510
pCL1920-Gfp-R	TCGTTTTATTTGATGCCTGGTTATTTGTATAGTTCATACATG		

pCL1920-F	CTAGTATTTCTCCTCTTTCT	pCL1920
pCL1920-R	CCAGGCATCAAATAAAACGA	vector
		fragment
pTrc-F	CCAGGCATCAAATAAAACG	pTrc99a vector
pTrc-R	GAGCTCGAATTCCATGGTC	fragment
pBBR-F	AGCTGTTTCCTGTGTGAAATTGTTATC	pBBR1MCS2
pBBR-R	CGTTAATATTTTGTTAAAATTC	vector
		fragment

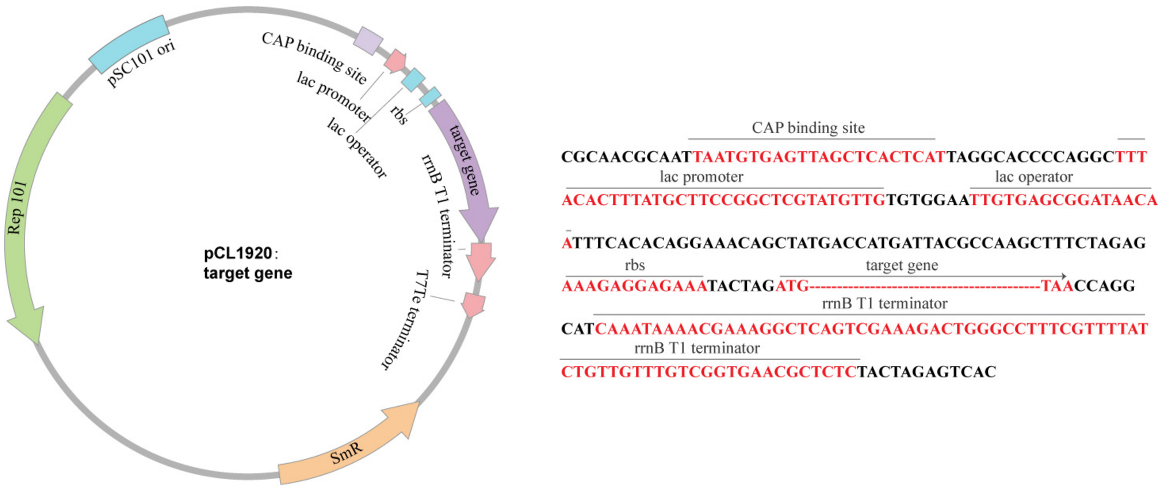

Figure S1. The construction of pCL1920 expression plasmids.

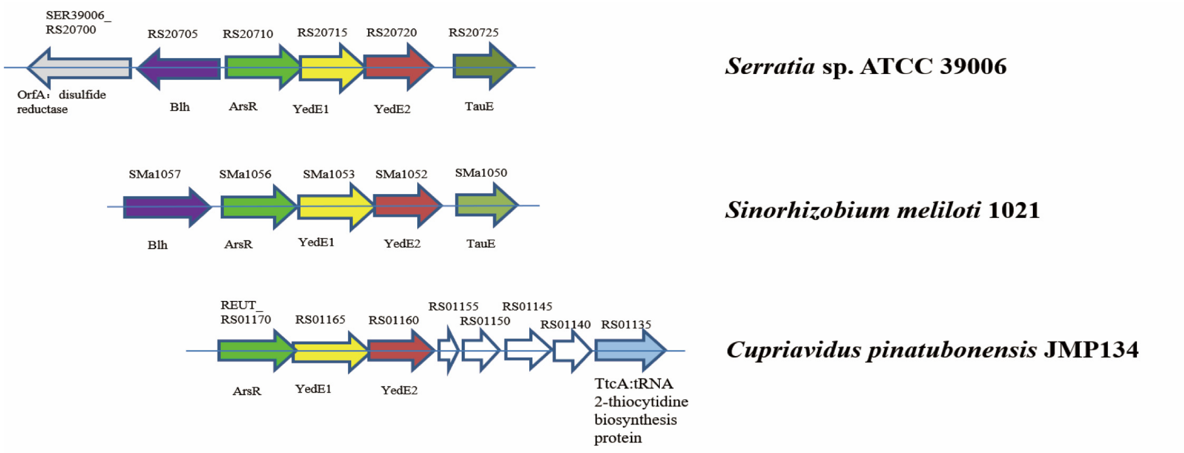

Figure S2. Gene Clusters of *yedE1*, *yedE2*, and sulfur metabolizing genes in three bacteria. Blh, persulfide dioxygenase; ArsR, ArsR family regulator; YedE, sulfur transporter; TauE; sulfite exporter. The locus tag of each gene is labelled above the gene.
